# Artificial intelligence in chorioretinal pathology through fundoscopy: a comprehensive review

**DOI:** 10.1186/s40942-024-00554-4

**Published:** 2024-04-23

**Authors:** Matthew Driban, Audrey Yan, Amrish Selvam, Joshua Ong, Kiran Kumar Vupparaboina, Jay Chhablani

**Affiliations:** 1 Department of Ophthalmology, University of Pittsburgh School of Medicine, Pittsburgh, PA USA; 2 Department of Medicine, West Virginia School of Osteopathic Medicine, Lewisburg, WV USA; 3 Michigan Medicine, University of Michigan, Ann Arbor, USA

**Keywords:** Artificial intelligence, Machine learning, Deep learning, Fundus, Fundoscopy, Choroid, Age-related macular degeneration, Diabetic retinopathy

## Abstract

**Background:**

Applications for artificial intelligence (AI) in ophthalmology are continually evolving. Fundoscopy is one of the oldest ocular imaging techniques but remains a mainstay in posterior segment imaging due to its prevalence, ease of use, and ongoing technological advancement. AI has been leveraged for fundoscopy to accomplish core tasks including segmentation, classification, and prediction.

**Main body:**

In this article we provide a review of AI in fundoscopy applied to representative chorioretinal pathologies, including diabetic retinopathy and age-related macular degeneration, among others. We conclude with a discussion of future directions and current limitations.

**Short conclusion:**

As AI evolves, it will become increasingly essential for the modern ophthalmologist to understand its applications and limitations to improve patient outcomes and continue to innovate.

## Background

Artificial intelligence (AI) is reshaping ophthalmology, especially in fundus imaging, aiding in segmentation, classification, and prediction of chorioretinal diseases like diabetic retinopathy (DR) and age-related macular degeneration (AMD). Fundus imaging is the technique of creating a two-dimensional representation of the three-dimensional semi-transparent retinal tissues using reflected yield [1–4]. Digital technology has supplanted stereo film, used for image capture, allowing for faster processing times and better image editing and acquisition [5, 6]. There are various subcategories within fundus imaging, such as stereo fundus, widefield fundus (WF), ultra-widefield fundus (UWF), fundus autofluorescence (FAF), color fundus (CF), standard fundus, and angiographic applications [1].

### Defining AI tasks in chorioretinal disease

AI is a powerful tool capable of learning a near universal set of tasks, but no single algorithm is universally successful. Algorithms are instead carefully designed to perform a specific task. AI in ophthalmology is most frequently used to automate three important tasks: image segmentation, classification, and prediction.

#### Segmentation

In the field of image processing, image segmentation refers to the partitioning of an image into multiple segments defined by a set of pixels. In medical imaging, segmentation plays an important role in identifying and highlighting regions of interest [7]. Classification and prediction models frequently use parameters and features obtained from analysis of regions of interest. As a result, segmentation is often performed as a first step in in the AI and image processing pipeline [8]. While this task could be performed using classic computer vision approaches such as tensor voting, AI-based learning techniques have shown great promise in improving accuracy and efficiency of segmentation.

In imaging of the chorioretinal space, segmentation plays a key role in delineating important structures, yielding biomarkers such as retinal thickness, intraretinal fluid volume, and choroidal thickness. In research, automated segmentation saves time and effort while improving accuracy. When dealing with large datasets, which are often required in DL tasks, automated segmentation is practically a necessity.

#### Classification

Classification is the task of assigning a category to a given input [9]. Classification is well-described problem not exclusive to AI. People classify objects regularly: for example, separating groceries into different food groups such as fruits, vegetables, breads, and meats. These groups are referred to as classes, categories, labels, or groups in AI. In ophthalmology, classifying a fundus image as polypoidal choroidal vasculopathy or age-related macular degeneration is a classification task [10]. Classification problems can be very specific, such as differentiating between stages of proliferative diabetic retinopathy or broad such as determining whether a patient needs referral or not [11]. These questions are of significant interest to clinicians as they can directly impact decision making. As a result, classification forms the backbone of AI use in medicine and is paving the way for automated diagnosis [12].

#### Prediction

Prediction is closely related to classification, but outputs a value or outcome rather than a category. Tasks that evaluate future outcomes, such as the chance of fluid recurrence or response to treatment, fall under the category of prediction. Models designed to solve prediction tasks may yield a continuous range of values: for example, the best-corrected visual acuity based on a set of clinical factors and treatment [13]. Even traditional classification models may be rethought as prediction models. Rather than discretely classifying AMD as intermediate or late stage, a prediction model may provide a continuous numeric value reflecting the severity of disease. Prediction models will serve as the foundation for automated assessment of disease progression and treatment outcomes.

## AI in chorioretinal pathology through fundoscopy

### Segmentation

Fundoscopy can visualize structures including retinal blood vessels, optic disc, optic cup, and macula [14]. Retinal blood vessel segmentation remains the key chorioretinal segmentation tasking using AI, along with the identification of chorioretinal lesions like microaneurysms, hemorrhages, and exudates [15]. However, there are relatively few articles regarding chorioretinal lesion segmentation using AI.

#### Segmentation of vessels

**Fig. 1:**
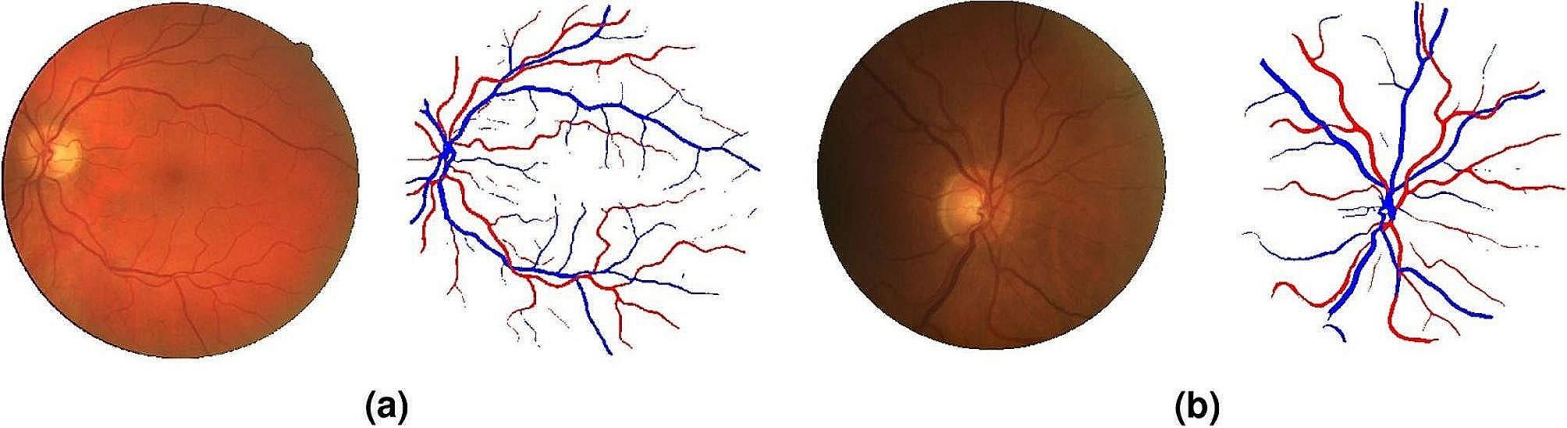
Fundus vessel segmentation using a W-Net tested on **a**) DRIVE and **b**) LES-AV datasets. Reprinted with permission from Galdran et al. Galdran, A., Anjos, A., Dolz, J. et al. State-of-the-art retinal vessel segmentation with minimalistic models. Sci Rep 12, 6174 (2022) under Creative Commons Attribution 4.0 International License (https://creativecommons.org/licenses/by/4.0/legalcode)

Retinal vessel segmentation is an important fundus task for the diagnosis and treatment of various ocular and cardiovascular pathologies [16] (Fig. 1).

Large datasets, such as DRIVE, STARE, and CHASE_DB1, have simplified vessel segmentation using machine learning (ML) and deep learning (DL) approaches, allowing for easy comparisons against other methods that have been similarly validated (Table 1) [17].

**Table 1:** Commonly Used Public Fundus Datasets for AI Applications

Dataset	Number of Images	Pathologies	Link
Digital Retinal Images for Vessel Extraction (DRIVE)	40	7	https://drive.grand-challenge.org/
Structured Analysis of the Retina (STARE)	397	13	http://cecas.clemson.edu/~ahoover/stare/
Child Hearth Health Study in England (CHASE_DB1)	28	Healthy	https://blogs.kingston.ac.uk/retinal/chasedb1/
High-Resolution Fundus Image Database (HRF)	45	DR, glaucoma	https://www5.cs.fau.de/research/data/fundus-images/
IOSTAR Retinal Vessel	30	N/A	http://www.retinacheck.org/download-iostar-retinal-vessel-segmentation-dataset
Standard Diabetic Retinopathy Database Calibration Level 0 (DIARETDB0)	130	DR	http://www.it.lut.fi/project/imageret/diaretdb0/
Standard Diabetic Retinopathy Database Calibration Level 1 (DIARETDB1)	89	DR	http://www.it.lut.fi/project/imageret/diaretdb1/index.html
Automated Retinal Image Analysis (ARIA)	143	AMD, DR	http://www.damianjjfarnell.com/?page_id=276
Age-Related Eye Disease Study 1/2 (AREDS1/2)	> 134,500	AMD, cataract	https://www.ncbi.nlm.nih.gov/projects/gap/cgi-bin/study.cgi?study_id=phs000001.v3.p1
Methods to Evaluate Segmentation and Indexing Techniques in the Field of Retinal Ophthalmology 1 (MESSIDOR1)	1,200	DR	https://www.adcis.net/en/third-party/messidor/
Methods to Evaluate Segmentation and Indexing Techniques in the Field of Retinal Ophthalmology 2 (MESSIDOR2)	1,748	DR	https://www.adcis.net/en/third-party/messidor2/
e-ophtha	463	DR	https://www.adcis.net/en/third-party/e-ophtha/

To our knowledge, Liskowski and Krawiec published the first example of AI segmentation of blood vessels from fundus images, achieving great success with a CNN validated of multiple datasets, including DRIVE, STARE, and CHASE_DB1 datasets [18]. This work paved the way for a significant rise in AI retinal vessel segmentation articles from 2017 to 2022.

The methods most used for retinal blood vessel segmentation include ML and DL techniques such as support vector machines (SVM), k-nearest neighbor, and U-net architecture [19–28]. Thin vessel problems are addressed by innovations such as SSANet and HHNet, which strike a balance between computing demands and accuracy [14, 29–35].

#### Segmentation of lesions

**Fig. 2:**
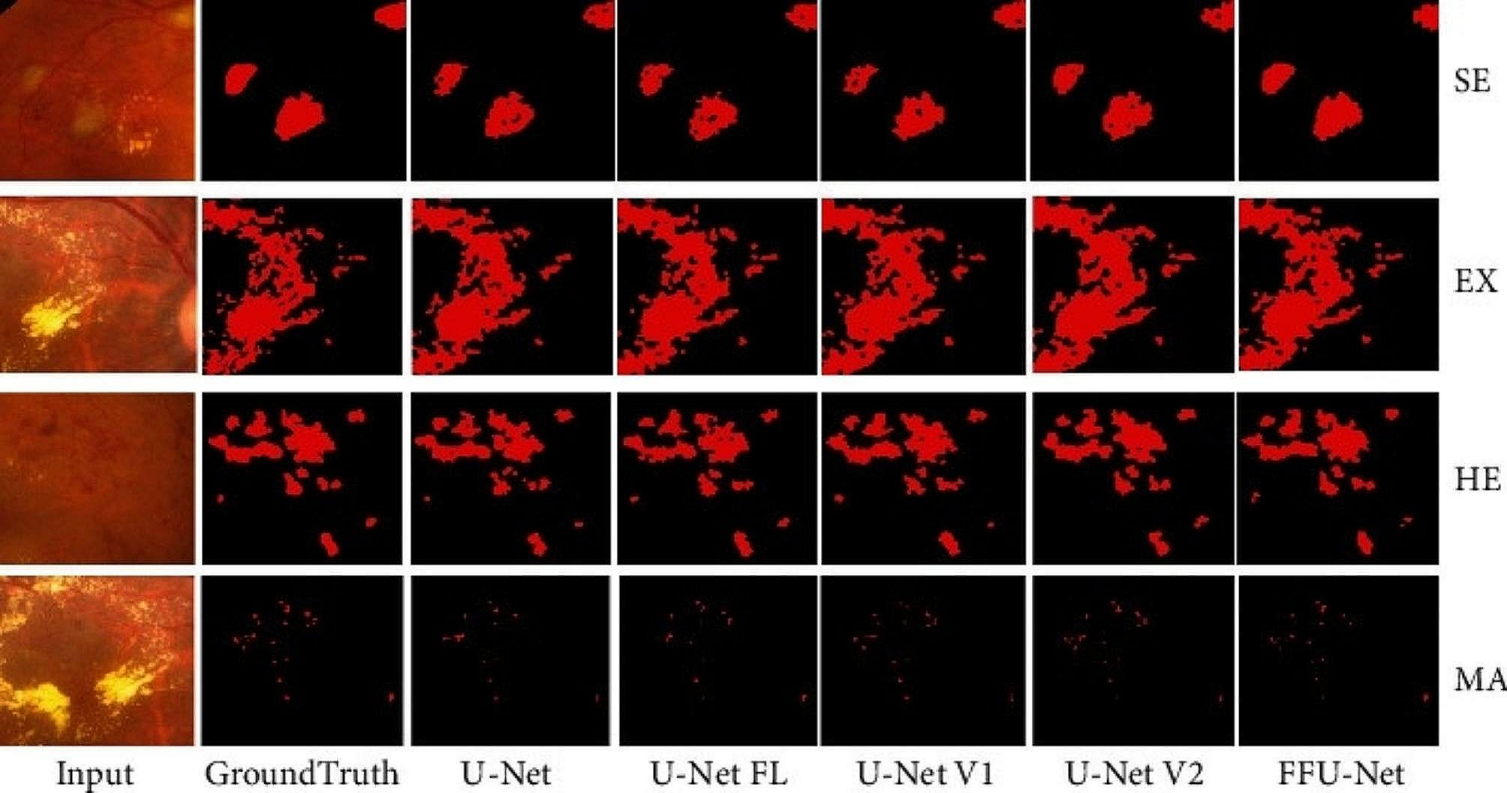
Automated segmentation of soft exudates (SE), hard exudates (EX), hemorrhage (HE), and microaneurysms (MA) using multiple U-Net architectures. Reprinted with permission from Xu et al. Xu Y, Zhou Z, Li X, Zhang N, Zhang M, Wei P. FFU-Net: Feature Fusion U-Net for Lesion Segmentation of Diabetic Retinopathy. Biomed Res Int. 2021 Jan 2;2021:6644071 under Creative Commons Attribution License (https://creativecommons.org/licenses/by/4.0/legalcode)

AI segmentation of lesions is an important, but less-investigated realm of chorioretinal fundus segmentation (Fig. 2). Four relevant studies were found (Table 2). Random forest, SVM, and CNN-based algorithms have been employed in research to segment a variety of findings, including geographic atrophy, drusen segmentation, macular edema, and retinal vein blockage [36–39].

**Table 2:** Lesion Segmentation with Fundus

Algorithm	Number of Articles	Results*	Groups
CNN	2	AUC:	Chen et al., Hassan et al.
		Acc: 0.94	
		Sens:	
		Spec:	
ML/Multimodal**	2	AUC:	Feeny et al., Khalid et al.
		Acc: 0.98	
		Sens: 1.00	
		Spec: 0.97	

*Average values across articles **ML algorithm(s) or combined use of ML and DL AUC, area under curve. *Acc*, accuracy. *Sens*, sensitivity. Spec, specificity

#### Utility of fundus segmentation

Fundus segmentation remains a key area of research for lesion diagnosis and biomarkers, even with ongoing advancements in algorithms for other imaging modalities. Recent studies have shown new therapeutic uses. OTNet, a CNN method with an AUC of 0.806 that grades arteriosclerosis based on retinal vascular segmentation, was described by Bai et al. [40]. AI’s usefulness in surgical settings was demonstrated by Xu et al.‘s description of an enhanced few-shot learning framework for accurate retinal vascular localization during central serous chorioretinopathy (CSCR) laser surgery [41]. As segmentation technology develops, real-time data may facilitate clinicians in carrying out essential tasks.

### Classification

AI classification of ocular pathologies from fundus images has focused on single and multiple pathologies. AMD and DR have received significant attention, with DL techniques prevailing.

#### Classification of a single pathology

Since the late 1990s, clinicians have been fascinated by automated classification of single diseases such as retinal vascular tortuosity and microaneurysms [42–44]. Commonly used datasets for validating classification algorithms include ARIA, AREDS1/2 for AMD, and MESSIDOR, DIARETDB0/1 for DR. STARE has been utilized for classification methods, as well as segmentation validation.

Many AI classifications of fundus images have been designed to diagnose AMD automatically (Table 3). Mookiah et al. published two of the first automatic AMD classification approaches from fundus images using a mixed methods approach, including decision tree, k-nearest neighbor, probabilistic neural network, and SVM [45, 46]. Their work achieved accuracies ranging from 90.19% to 97.78%, with later improvements achieving 100% accuracy via Locality Sensitive Discriminant Analysis.

**Table 3:** AMD Classification with Fundus

Algorithm	Number of Articles	Results*	Groups
CNN	5	AUC: 0.95	Burlina et al., Chen et al. (a), Chen et al. (b), Keel et al., Matsuba et al.
		Acc: 0.54	
		Sens: 0.98	
		Spec: 0.97	
ML/Multimodal**	5	AUC: 0.93	Acharya et al., Govindaiah et al., Mookiah et al. (a), Mookiah et al. (b), Yoo et al.
		Acc: 0.96	
		Sens: 0.90	
		Spec: 0.98	

*Average values across articles **ML algorithm(s) or combined use of ML and DL AUC, area under curve. *Acc*, accuracy. *Sens*, sensitivity. Spec, specificity

CNNs like ResNet, Inception-ResNet-V2, and DeepSeeNet have achieved great accuracies in AMD classification, with AUCs above 0.970 [47–50]. Innovative techniques such as UWF-based CNN and multimodal frameworks improve AMD detection, resulting in impressive AUCs [51, 52]. 

DR has historically been a secondary emphasis of AI classification of a single pathology (Table 4). DL techniques, particularly, IDx-DR X2.1, result in FDA-approved AI diagnostic systems, while Gayathri et al. use ML approaches to obtain high precision and recall [53–58].

**Table 4:** DR Classification with Fundus

Algorithm	Number of Articles	Results*	Groups
CNN	8	AUC: 0.97	Abràmoff et al. (a), Abràmoff et al. (b), Gargeya et al., Gulshan et al., Singh and Gorantla, Tang et al., Ting et al., Zhang et al.
		Acc: 0.98	
		Sens: 0.91	
		Spec: 0.93	
ML/Multimodal**	5	AUC: 0.95	Cao et al., Gayathri et al., Long et al., Yu et al., Zhang et al.
		Acc: 0.95	
		Sens: 0.90	
		Spec: 0.96	

*Average values across articles **ML algorithm(s) or combined use of ML and DL AUC, area under curve. *Acc*, accuracy. *Sens*, sensitivity. Spec, specificity

Cao et al. published the first paper focusing on AI detection of microaneurysms, critical for early DR diagnosis with an AUC of 0.985 [59]. Yu et al. and Tang et al. classified neovascularization with 95.23% and 99.48% accuracy, respectively [60, 61]. In DR, Sahlsten et al’s Inception-v3 model detected macular edema with an AUC of 0.987 [62]. Singh and Gorantla’s DMENet attained 96.12% accuracy in early macular edema identification [63].

In addition to AMD and DR, several other pathologies have been classified using AI on fundus photos (Table 5). For pathologic myopia (PM), Lu et al. and Rauf et al. developed CNN techniques that achieved similar AUCs of 0.979 and 0.9845, respectively [64, 65] Du et al. performed a similar feature-based classification for PM and achieved a high overall detection rate of 92.08%, demonstrating AI’s potential for identifying various PM lesions [66].

**Table 5:** Classification of Non-AMD, Non-DR Pathologies with Fundus

Algorithm	Number of Articles	Results*	Groups
CNN	7	AUC: 0.97	Brown et al., Cai et al., Du et al., Lu et al., Lu et al., Rauf et al., Zhen et al.
		Acc: 0.97	
		Sens: 0.82	
		Spec: 0.96	
ML/Multimodal**	0		

*Average values across articles **ML algorithm(s) or combined use of ML and DL AUC, area under curve. *Acc*, accuracy. *Sens*, sensitivity. Spec, specificity

Zhen et al. identified CSCR using Inception-V3, achieving AUC 0.934 [67]. Brown et al. used two CNNs, Inception-v1 and U-Net, for diagnose of plus disease in retinopathy of prematurity, with a 100% sensitivity and 94% specificity [68]. Cai et al. used Inception-v3 to automatically classify sea fan neovascularization in sickle cell hemoglobinopathy patients, attaining an AUC of 0.988, sensitivity of 97.4%, and specific of 97.0% [69].

#### Classification of multiple pathologies

Since 2017, multi-pathology classification using ML and DL has gained popularity, with the goal of rapidly detecting many diseases from a single image, which is critical for new patients. The first such study classified imagines into 10 retinal diseases using DL models like VGG-19, with VGG-19 transfer learning-random forest surpassing the others [70]. While a promising study on multiple disease classification, the authors acknowledged that their pilot study did not fully demonstrate the benefits of using DL for this task, owing to the limited sample size.

For the rest of this section, we will go over additional attempts at multiple disease classification utilizing a range of AI algorithms for many diseases (Table 6).

**Table 6:** Classification of Multiple Pathologies with Fundus

Algorithm	Number of Articles	Results*	Groups
CNN	10	AUC: 0.97	Cen et al., Choi et al., Chou et al., González-Gonzalo et al., Keel et al., Kim et al., Sahlsten et al., Son et al., Xu et al., Yu-Chuan Kang et al.
		Acc: 0.724	
		Sens: 0.89	
		Spec: 0.94	
ML/Multimodal**	6	AUC: 0.89	Antaki et al., Balasubramanian and Ananthamoorthy, Koh et al., Porwal et al., Standardization of Uveitis Nomenclature (SUN) Working Group, Tan et al.
		Acc: 0.95	
		Sens: 0.88	
		Spec: 0.93	

*Average values across articles **ML algorithm(s) or combined use of ML and DL AUC, area under curve. *Acc*, accuracy. *Sens*, sensitivity. Spec, specificity

**Fig. 3:**
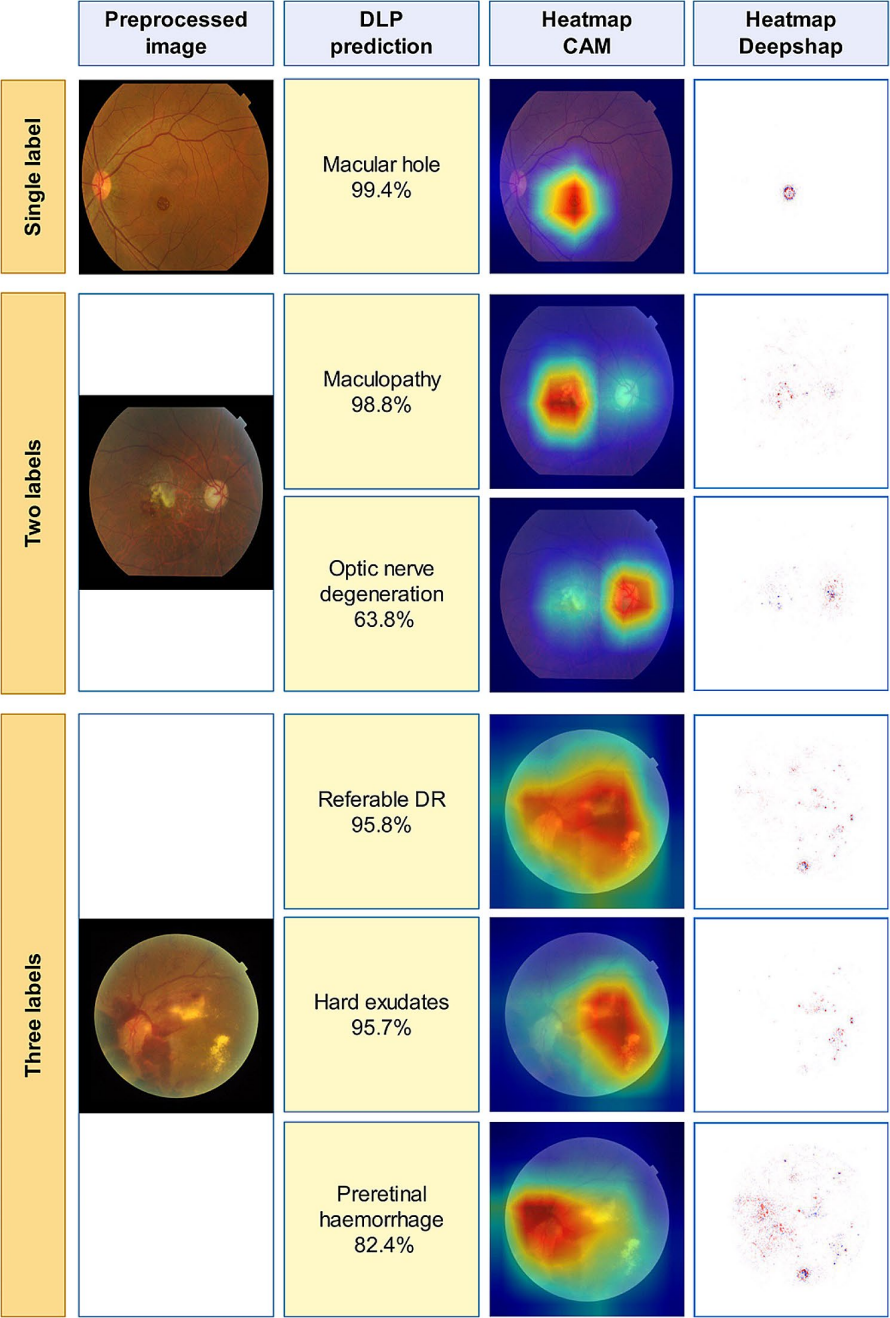
Representative heatmap and feature detection for classification of multiple retinal pathologies. Reprinted with permission from Cen et al. Cen, LP., Ji, J., Lin, JW. et al. Automatic detection of 39 fundus diseases and conditions in retinal photographs using deep neural networks. Nat Commun 12, 4828 (2021) under Creative Commons Attribution License (https://creativecommons.org/licenses/by/4.0/legalcode)

We found two algorithms for classification of AMD and DR from normal images. The first used multiple AI algorithms, including AdaBoost, c4.5, logistic regression, naive bayes, neural network, random forest, SVM [71]. The random forest classifier outperformed other methods with an AUC exceeding 0.995 and the authors contend that their approach is feasible even with a small image pool. González-Gonzalo et al. used RetCAD v.1.3.0 to attain AUC values of 95.1% for DR and 94.9% for AMD [72]. Studies also explored glaucoma classification alongside AMD-DR detection and focused on distinguishing between AMD and polypoidal choroidal vasculopathy (PCV), along with identifying less frequent pathologies and myopic conditions [73–77]. CNN-based approaches have been suggested for detecting significant findings in retinal images and classifying nine posterior segment pathologies [78–83] Cen et al. developed a CNN capable of detecting 39 common retinal diseases, showcasing AI’s potential in ophthalmologic practices [84] (Fig. 3).

#### Classification of stages in a pathology

AI staging of chorioretinal pathologies has focused on AMD, DR, and a few additional pathologies (Table 7). Burlina et al. pioneered AMD grading with DL models like AlexNet and OverFeat, achieving encouraging results but falling short of human grading [85–87].

**Table 7:** Classification of Stages in a pathology with fundus

Algorithm	Number of Articles	Results*	Groups
CNN	8	AUC: 0.96	Alyoubi et al., Burlina et al. (a), Burlina et al. (b), Campbell et al., Heo et al., Peng et al., Shaban et al., Wan et al.
		Acc: 0.82	
		Sens: 0.88	
		Spec: 0.95	
ML/Multimodal**	4	AUC:	Akbar et al., Grassman et al., Hosoda et al., Murugeswari and Sukanesh
		Acc: 0.93	
		Sens:	
		Spec:	

*Average values across articles **ML algorithm(s) or combined use of ML and DL AUC, area under curve. *Acc*, accuracy. *Sens*, sensitivity. Spec, specificity

**Fig. 4:**
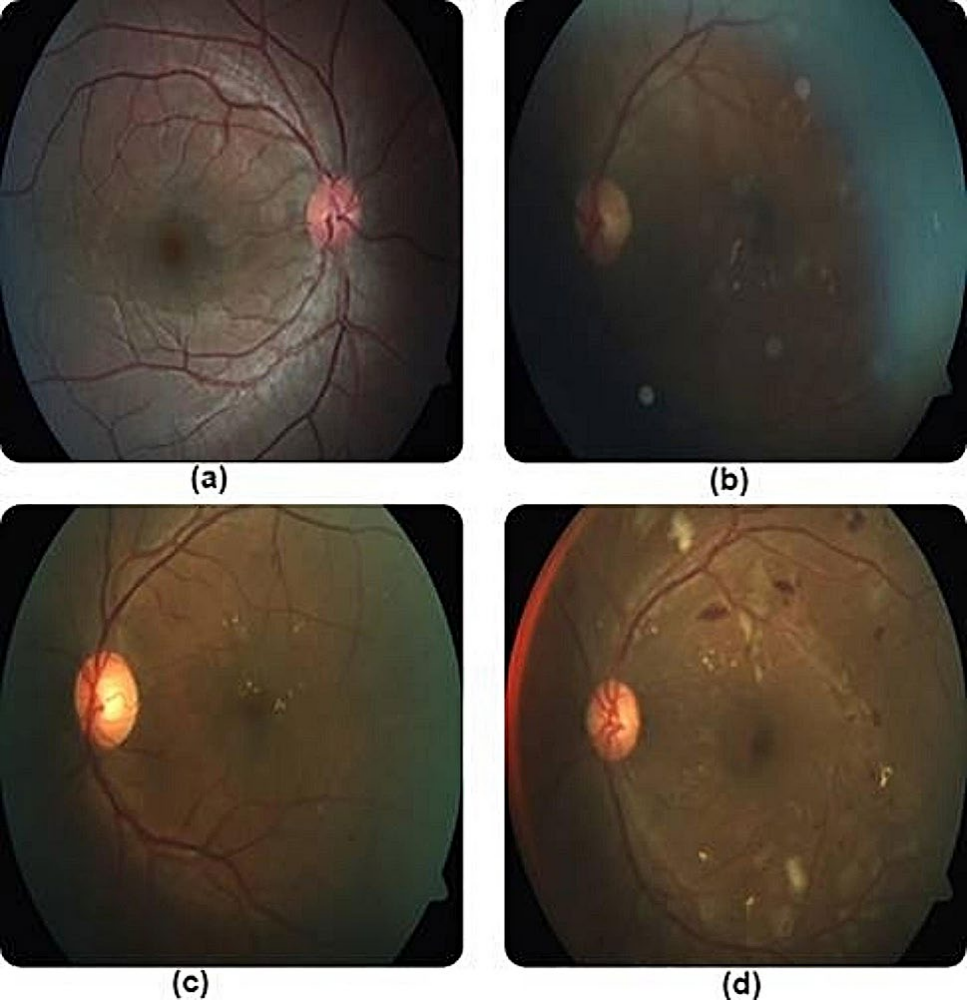
Misclassified images of DR in multiple stages due to poor lighting and contrast. Reprinted with permission from Shaban et al. Shaban M, Ogur Z, Mahmoud A, Switala A, Shalaby A, Abu Khalifeh H, Ghazal M, Fraiwan L, Giridharan G, Sandhu H, El-Baz AS. A convolutional neural network for the screening and staging of diabetic retinopathy. PLoS One. 2020 Jun 22;15 [6]:e0233514 under Creative Commons Attribution License (https://creativecommons.org/licenses/by/4.0/legalcode)

Grassmann et al. used six CNNs (AlexNet, GoogLeNet, VGG, Inception-v3, ResNet, and Inception-ResNet-v2) to grade AMD with 63.3% accuracy [88]. DeepSeeNet outperformed physicians in identifying large drusen and pigmentary abnormalities in AMD, but was inferior in detecting late AMD (stage 5) [89]. VGG-16 identified wet vs. dry AMD with higher accuracy than first-year residents [90]. K-means cluster analysis identified pachychoroid features associated with improved visual acuity in AMD patients [91]. Despite their variable accuracy, AI algorithms demonstrated potential in a variety of classification systems. SVM achieved 98.33% accuracy in DR staging by analyzing fundus and OCT images [92]. CNNs achieved 88–89% accuracy in classifying DR severity, addressing issues like poor image quality and overfitting [93] (Fig. 4).

CNN512 and YOLOv3 achieved 89% accuracy in DR staging [11]. AI was also employed in assessing myopia risk, hypertensive retinopathy, and retinopathy of prematurity with high accuracies exceeding 95% in various modules and datasets, demonstrating AI’s potential in grading diverse eye pathologies [94–96].

### Prediction

In fundoscopy, prediction is used to predict future occurrences, such as the disease course, which is important for treatment planning and follow-up. The goal of five AI prediction experiments using fundus pictures was to forecast the progression of DR or AMD (Table 8). AI prediction in fundoscopy has demonstrated promise in improving ocular prognostics, despite being a relatively new subject.

**Table 8:** Prediction with fundus

Algorithm	Number of Articles	Results*	Groups
CNN	3	AUC: 0.84	Arcadu et al., Hua et al., Peng et al.
		Acc: 0.89	
		Sens: 0.97	
		Spec: 0.82	
ML/Multimodal**	2	AUC:	Bhuiyan et al., Govindaiah et al.
		Acc: 0.81	
		Sens: 0.83	
		Spec: 0.80	

*Average values across articles **ML algorithm(s) or combined use of ML and DL AUC, area under curve. *Acc*, accuracy. *Sens*, sensitivity. Spec, specificity

**Fig. 5:**
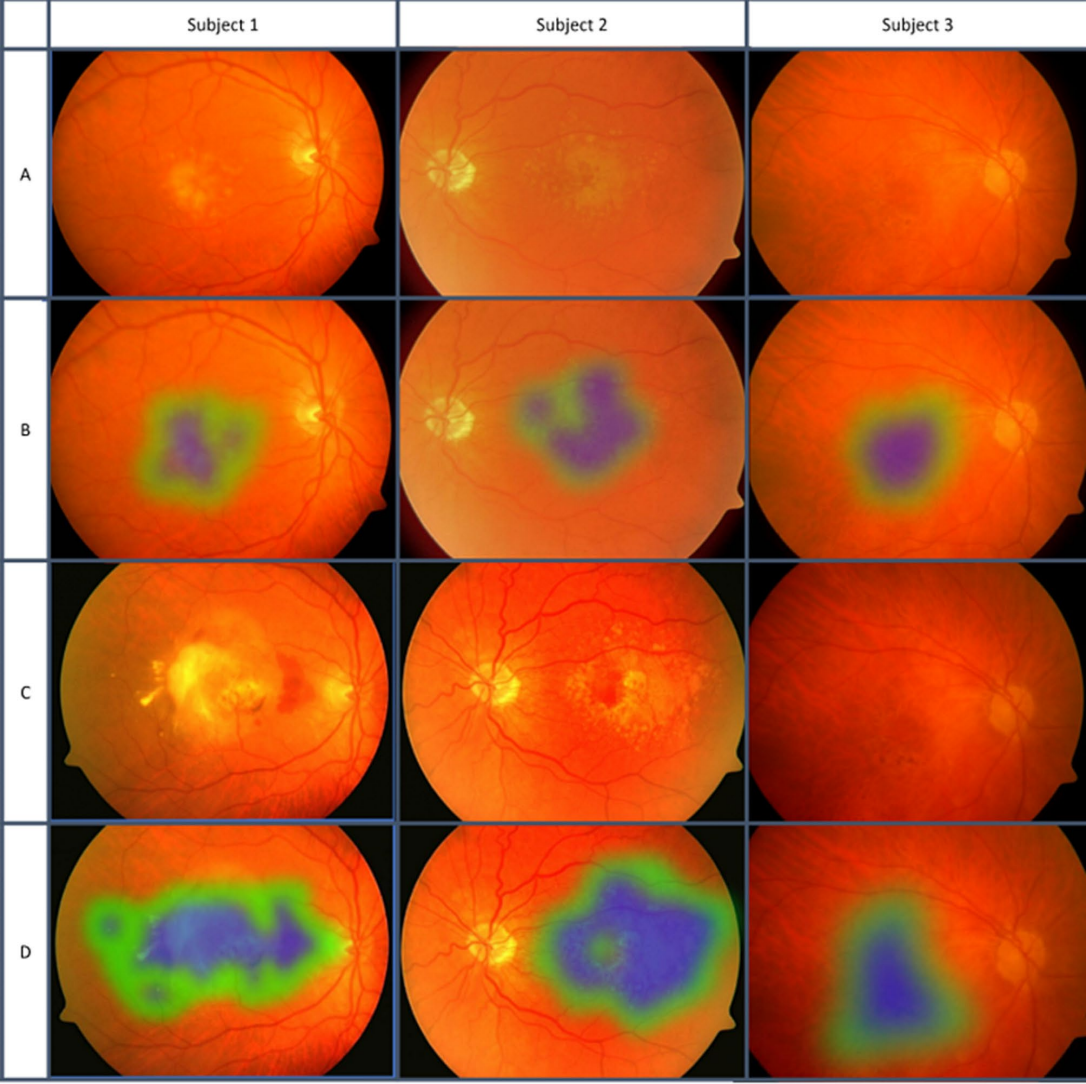
Classification of stages of AMD. Blue represents strong signs of AMD, while green represents weaker signs of AMD. Larger areas with more blue resulted in classification into a later stage. Reprinted with permission from Bhuiyan et al. Alauddin Bhuiyan, Tien Yin Wong, Daniel Shu Wei Ting, Arun Govindaiah, Eric H. Souied, R. Theodore Smith; Artificial Intelligence to Stratify Severity of Age-Related Macular Degeneration (AMD) and Predict Risk of Progression to Late AMD. Trans. Vis. Sci. Tech. 2020;9 [2]:25 under Creative Commons Attribution-NonCommercial-NoDerivatives 4.0 International License (https://creativecommons.org/licenses/by-nc-nd/4.0/legalcode)

Arcadu et al. and Hua et al. employed DL to predict DR risk, with AUCs of 0.79 and 88.8%, respectively [97, 98]. The first example of AMD development was published in 2020 by Bhuiyan et al., who distinguished between early/none and intermediate/late AMD with 99.2% accuracy [99] (Fig. 5). Their two-year prediction model exhibited an overall accuracy of 86.36% for late-stage AMD progression. While this model performed well in predicting AMD in general, it was not as successful in differentiating between the wet and dry subtypes.

Peng et al. and Govindaiah et al. developed AI approaches for accurately predicting late AMD, highlighting AI’s potential to improve prognostics for eye disorders [100, 101].

## Discussion

AI is transforming ophthalmology, particularly in forecasting disease progression like AMD and CSCR and treatment outcomes [102–106]. Despite challenges such as inconsistent therapy response, AI can enhance results and save costs. Enhancing OCT picture quality by denoising algorithms and image augmentation aids in diagnosis and therapy planning.

New developments in AI, such vision transformers (ViT), are broadening the range of uses for image processing. Convulsion layers have shown to be a particularly successful way for ViTs to incorporate image patches; in 2021, ViT-G beat previous models on the ImageNet dataset [107]. However, ViT networks pose difficulties for applications such as OCTs, as they demand large datasets and substantial processing resources [108]. Generative adversarial networks (GAN) are another type of deep learning technology that is becoming more and more popular. GAN is capable of image synthesis, superresolution, and picture-to-image translation [109, 110]. Deep convolutional GANs, or DCGANs, train the operators within CNNs, whereas conditional GANs (C-GANs) supply extra data to improve created data representations [111–113]. Zhang et al. highlighted the value of GANs in chorioretinal research by using them to remove retinal shadows and improve choroid area imaging [114].

AI in fundoscopy bridges access barriers to expensive imaging modalities because fundus images anticipate OCT biomarkers and offer angiographic images [115]. Although the creation of AI is made easier by automated machine learning (AutoML), high-quality datasets are still hard to come by. Federated learning and cooperative efforts may be able to solve this problem, increasing the application of AI in healthcare.

While many of AI’s applications in ophthalmology have been image-based to date, the rapid development and acceptance of large language models (LLMs), including, most famously, ChatGPT (OpenAI, San Francisco, USA), heralds an approaching era in which text-based generative algorithms are ubiquitous in clinical and research contexts [116]. LLMs offer the potential to guide clinical decision making for physicians, help patients self-triage and self-diagnose, generate novel research ideas for clinician-scientists, and assist in training the next generation of ophthalmologists, among other powerful benefits [117]. It should be noted that, like many AI applications across medicine and society, LLMs posit important ethical and implementation challenges alongside their potential to optimize clinical decision making and improve patient experiences. These include privacy concerns, especially for models trained using electronic medical record data, false/misleading responses, lack of accessible data to train a model specifically designed for ophthalmologic purposes, and other ethical concerns [118]. In other words, while LLMs will almost certainly find some role in clinical and investigative ophthalmology, and in fact have already seen preliminary studies explored, their use need be evaluated carefully to ensure continued quality and integrity in ophthalmic care [119].

## Conclusions

Fundoscopy, despite its age and limitations, remains a valuable tool to image the posterior segment. While many AI applications in fundoscopy are still nascent, it is only a matter of time before these algorithms become commonplace in clinical and research settings. With innovation, acceptance, and understanding from modern day clinicians, our ability to treat and diagnose chorioretinal pathology is set to continue to improve in the golden age of AI.

## Data Availability

Not applicable.
